# Garrord on Scurvy

**Published:** 1848

**Authors:** A. B. Garrod

**Affiliations:** Assistant Physician to University College Hospital, London


### ARTICLE X.

*Scurvy.* By DR. A. B. GARROD, Assistant Physician to University College Hospital, London.

[It seems to have been fully proved that whatever circumstances may influenc the production of scurvy, its immediate cause is to be sought for in the nature of the food; in the absence or deficiency of one or other of its *organic* or *inorganic* constituents. Dr. Garrod points out many objections to the opinion that a deficiency of any of the *organic* constituents of the food produces scurvy; and states that from minute researches into the composition of scorbutic articles of diet, and into the state of the blood in scorbutic patients, he has been led to the following conclusions:]

1st. That in all scorbutic diets, *Potash* exists in much smaller quantities than in those which are capable of maintaining health. 2d. That all substances proved to act as anti scorbutics contain a large amount of *Potash*. 3d. That in scurvy the blood is deficient in *Potash*, and the amount of that substance thrown out by the kidneys less than that which occurs in health. 4th. That scorbutic patients will recover when *Potash* is added to their food, the other constituents remaining as before, both in quantity and quality, and without the use of suculent vegetables or milk. 5th. That the theory which ascribes the cause of scurvy to a deficiency of *Potash* in the food, is also capable of rationally explaining many symptoms of that disease.

[The first proposition is proved by an examination of the dietary of the Credition Union, (*see Prov. Med. and Surg. Jour., June*, 1847). After giving a statement of the amount of potash contained in various articles of food, Dr. Garrod says,]

If we estimate the amount of potash taken by the inmates of this work-house, we shall find the men's food to contain about 186 grains, and the women's about 181 grains. This amount would be much influenced by the mode in which the potatoes were cooked; if not too much boiled, and with the skins on, they would contain a much larger amount of potash than if boiled without their skins, and much done. Under this diet the inmates remained healthy; but, owing to the scarcity of potatoes, boiled rice in equal weights was substituted, and in a few months the inmates became scorbutic. When the change was made, the weekly amount of potash taken by the men was about 51 grains, and by the women 46 grains, or a reduction of more than two-thirds took place. Rice and potatoes do not differ much in their composition, except in the salt of *potash* contained by the latter. Both contain starch and vegetable albumen. In the weekly diet list for the military prisoners at the Milbank Penitentiary, when they were subject to scurvy (see Dr. Baly's paper in *London Medical Gazette*, Vol. I. 1841-2,) we find the amount of potash taken by each prisoner during the first three months of imprisonment to be about 44 grains; during the second three months, about 50 grains; after six months, about 68 grains. At present, when potatoes are added, the weekly amount of potash is from 210 to 230 grains, and no case of scurvy has arisen since the change.

[Several other confirmatory facts are adduced, and Dr. Garrod proceeds to show that,]

*All bodies proved to be anti-scorbutic contain a large amount of potash.* All fruits contan this substance in abundance, as oranges, lemons, limes, grapes, gooseberries, &c., and these are all highly anti-scorbutic. Potatoes also, which perhaps are the most valuable as an addition to a dietary for the purpose of preventing scurvy, and owing to the scarcity of which article this disease has been so prevalent within the last two years, contain, as the above analyses prove, a very large amount of potash, and when boiled (not too much, and unpeeled,) still retain most of that ingredient; this also accords with the fact, that potatoes, when cooked in the ordinary way, are anti-scorbutic, and at the same time explains why the hard core of that tuber, which is so much liked by the Irishmen, is most powerful in preventing the occurrence of scurvy.

[Milk and fresh meat, cabbages, onions, turnips, spruce beer, &c., also contain considerable quantities of potash. As to the condition of the blood, Dr. Garrod found that the blood of a scorbutic contained little more than one-third of the amount of potash that is contained in healthy blood. To come to the practical application of these facts, Dr. Garrod shows that,]

*Scorbutic patients, when kept under a diet which gave rise to the disease, recover, when a few grains of Potash are added to their food.* In several cases which came under my care, the treatment consisted in the daily administration of a few grains (from 12 to 20,) of some salt of potash, mixed with syrup of water. Sometimes the bitartrate, at other times the acetate, and also the carbonate and phosphate were used. All the salts appeared to act alike, and I have little doubt the chloride of potassium would be found equally efficacious. When the cases were thus treated, all vegetables, milk, and malt liquors were strictly prohibited; and yet the patients rapidly recovered. Other cases were treated by fresh vegetables and milk; these also recovered, but certainly not more quickly than those from whom these substances were withheld, and potash salts substituted. On looking over the works of several writers on scurvy, I have frequently found that some *Potash* salt has been administered with *marked* benefit; thus, nitre has been recommended, nitre dissolved in vinegar, the bitartrate of potash, the oxalate of potassa; but the efficacy has always been ascribed to the acid contained in these substances, and no attention has been paid to the base.

*The theory which ascribes the cause of scurvy to a deficiency of Potash in the system, is capable of explaining some of its symptoms.* Both soda and potash are constant constituents of the animal body, and it appears that they are not capable of replacing each other; for example, we always find the potash to exist in large quantities in the ash of muscle, soda in very small quantities (Berzelius, Liebig); in the ash of the blood we find the relation reversed. It appears also, that the muscular system requires the presence of potash, and we should therefore expect to find that where there is a deficient supply of this base, the effect would soon be manifested in the functions of that system. This we find to be the case in scurvy; without any amount of wasting of the body, we find marked muscular debility, and this perhaps is one of the earliest symptoms of the disease.

*Conclusion.* I have ventured to make public this theory of the cause and nature of scurvy sooner than I otherwise, wished both on account of the difficulty of procuring cases of this disease at the present time, and from the conviction that its being made known to the profession at large would be the most ready mode of having it confirmed or disproved. If true, it will be seen at once that its applications will be of the utmost importance, and the occurrence of scurvy, both at sea and on land, can be most readily prevented, by the introduction of a few grains of some potash salt, as the phosphate, chloride, tartrate, &c., or by these being taken in a separate state. At sea, its application would be invaluable, from the cheapness, stability, and the small space occupied by the remedy, when compared with lime juice; from its being at all times to be procured from the ashes of wood or plants, especially tobacco, which contains it in abundance. If found to be a mere hypothesis, I have this apology to make, that in my mind it accounts better for the occurrence of this disease than any other yet offered; and it will still be an interesting fact, that *Potash* always accompanies the *real* antiscorbutic principle, was found deficient in scorbutic blood, and that several cases of scurvy rapidly recovered under the use of some of its salts, without the administration of any other remedy, dietetical or medicinal.—*Monthly Jour. of Med. Science*, Braith's Ret.

